# Carbohydrate sulfotransferases: a review of emerging diagnostic and prognostic applications

**DOI:** 10.11613/BM.2023.030503

**Published:** 2023-08-05

**Authors:** Gramos Begolli, Ivana Marković, Jelena Knežević, Željko Debeljak

**Affiliations:** 1 Clinic of medical biochemistry, University clinical center of Kosovo, Prishtina, Kosovo; 2 Clinical institute of laboratory diagnostics, University hospital centre Osijek, Osijek, Croatia; 3 Faculty of medicine, Josip Juraj Strossmayer University of Osijek, Osijek, Croatia; 4 Laboratory for advanced genomics, Ruđer Bošković Institute, Zagreb, Croatia; 5 Faculty for dental medicine and health, Josip Juraj Strossmayer University of Osijek, Osijek, Croatia

**Keywords:** cancer, carbohydrate sulfotransferases, connective tissue, inflammation, tumour markers

## Abstract

Carbohydrate sulfotransferases (CHST) catalyse the biosynthesis of proteoglycans that enable physical interactions and signalling between different neighbouring cells in physiological and pathological states. The study aim was to provide an overview of emerging diagnostic and prognostic applications of CHST. PubMed database search was conducted using the keywords “carbohydrate sulfotransferase” together with appropriate inclusion and exclusion criteria, whereby 41 publications were selected. Additionally, 40 records on CHST genetic and biochemical properties were hand-picked from UniProt, GeneCards, InterPro, and neXtProt databases. Carbohydrate sulfotransferases have been applied mainly in diagnostics of connective tissue disorders, cancer and inflammations. The lack of CHST activity was found in congenital connective tissue disorders while CHST overexpression was detected in different malignancies. Mutations of *CHST3* gene cause skeletal dysplasia, chondrodysplasia, and autosomal recessive multiple joint dislocations while increased tissue expression of *CHST11*, *CHST12* and *CHST15* is an unfavourable prognostic factor in ovarian cancer, glioblastoma and pancreatic cancer, respectively. Recently, *CHST11* and *CHST15* overexpression in the vascular smooth muscle cells was linked to the severe lung pathology in COVID-19 patients. Promising CHST diagnostic and prognostic applications have been described but larger clinical studies and robust analytical procedures are required for the more reliable diagnostic performance estimations.

## Introduction

Sulfation of small molecules, carbohydrates and proteins, is a set of metabolic reactions that occur in most organisms. This important modifying process takes place in bacteria, plants, and mammals. Sulfation is linked to a number of cellular signalling events and receptor-ligand binding modifications associated with physiological and pathological processes such as hormone regulation, cartilage creation, cancer cell spreading and solubilisation of harmful xenobiotics (*1*). In addition, sugar sulfation has also been shown to affect leukocyte-endothelial cell adhesion at chronic inflammation sites and expansion of neurons and astrocytes (*2*). Sulfations are catalysed by sulfotransferases (SULT), enzymes that are primarily responsible for the transfer of sulphate from a donor molecule, 3’-phosphoadenosine-5’-phosphosulfate (PAPS), to various hydroxyl and amine substrates (*3*). Two classes of SULT have been described: cytosolic SULT and membrane-associated SULT. Cytosolic SULT are involved in the sulfation of low-molecular-mass endogenous and exogenous compounds such as hormones, bioamines, drugs and other xenobiotic agents (*4*). On the other hand, glycosaminoglycans (GAG), proteoglycans (PG), and glycolipids that mediate processes like carcinogenesis and cartilage formation are created mainly by carbohydrates sulfation that are catalysed by the membrane-associated SULT (*5*). Carbohydrate sulfotransferases (CHST) are Golgi network localized membrane associated enzymes. The glycans associated with lipids and proteins moving through the secretory pathway are their primary substrates. The extracellular matrix (ECM) is made up largely of GAG and PG, which are particularly abundant in connective tissues (*6*). Glycosaminoglycans are linear acidic polysaccharides of heparin/heparan sulphate, chondroitin/dermatan sulphate (CS), keratan sulphate (KS) or hyaluronic type (*7*). Depending on the attached GAG chains and relative size, PG are categorized into plasma membrane associated heparan sulphate proteoglycans (HPSPG), chondroitin or dermatan sulphate proteoglycans (CSPG and DSPG) and keratan sulphate proteoglycans (KSPG). Versican, aggrecan and brevican represent some ECM-forming CSPG while biglycan and decorin are ECM-forming DSPG. Fibromodulin is a member of KSPG family which is also involved in the ECM formation by participating in the collagen assembly. Interestingly, members of one PG family, syndecans, contain heparan sulphate, dermatan sulphate and chondroitin sulphate GAG chains. As part of ECM, CSPG and DSPG interact with cells, including the inflammatory cells, either indirectly *via* hyaluronic and other GAG or directly *via* receptors such as CD44 antigen which is also CSPG (*8*). These interactions enable a specific form of communication and connection between neighbouring cells. In addition to KSPG biosynthesis, CHST catalyse CSPG biosynthesis and thus enable formation of connections or, indirectly, transmit critical signals between neighbouring cells in physiological and pathological conditions (*9*). The CHST nomenclature and their enzymatic properties are summarized in Table 1.

**Table 1:** Nomenclature and enzymatic properties of carbohydrate sulfotransferases

**Name (synonym)**	**Catalysed reaction**	**Reference**
CHST1 (Keratansulfate Gal-6 sulfotransferase)	Sulphate transfer to position 6 of internal galactose (Gal) residues of keratan	(*14*-*17*)
CHST2 (N-acetylglucosamine 6-O-sulfotransferase 1)	Sulphate transfer to position 6 of non-reducing GlcNAc residues within keratan-like structures on N-linked glycans and within mucin-associated glycans	(*18*-*20*)
CHST3 (Chondroitin 6-0-Sulfotransferase 1)	Sulphate transfer to position 6 of GalNAc residue of chondroitin	(*21*-*23*)
CHST4 (Galactose/N-acetylglucosamine/N-acetylglucosamine 6-O-sulfotransferase 3)	Sulphate transfer to the hydroxyl group at C-6 position of the non-reducing GlcNAc residue within O-linked mucin-type glycans	(*24*, *25*)
CHST5 (N-acetylglucosamine 6-O-sulfotransferase 3)	Sulphate transfer to position 6 of non- GlcNAc residues and O-linked sugars of mucin-type acceptors	(*26*-*30*)
CHST6 (Corneal N-acetylglucosamine-6-O-sulfotransferase)	Sulphate transfer to position 6 of non-reducing GlcNAc residues of keratan	(*31*-*33*)
CHST7 (Chondroitin 6-sulfotransferase 2)	Sulphate transfer to position 6 of non- GlcNAc residues and to position 6 of the GalNAc residue of chondroitin	(*34*-*36*)
CHST8 (GalNAc-4-O-sulfotransferase 1)	Sulphate transfer to position 4 of non-reducing GalNAc residues in both N-glycans and O-glycans	(*37*, *38*)
CHST9 (GalNAc-4-O-sulfotransferase 2)	Sulphate transfer to position 4 of non-reducing GalNAc residues in both N-glycans and O-glycans	(*39*, *40*)
CHST10 (Human Natural Killer-1 sulfotransferase)	Sulphate transfer to position 3 of terminal glucuronic acid of both protein- and lipid-linked oligosaccharides	(*41*-*43*)
CHST11 (Chondroitin 4-O-sulfotransferase 1)	Sulphate transfer to position 4 of GalNAc residue of chondroitin	(*44*, *45*)
CHST12 (Chondroitin 4-O-sulfotransferase 2)	Sulphate transfer to position 4 of GalNAc residue of chondroitin and desulfated dermatan sulphate	(*46**, **47*)
CHST13 (Chondroitin 4-O-sulfotransferase 3)	Sulphate transfer to position 4 of GalNAc residue of chondroitin	(*48*, *49*)
CHST14 (Dermatan 4-sulfotransferase 1)	Sulphate transfer to position 4 of GalNAc residue of dermatan sulphate	(*50*, *51*)
CHST15 (N-acetylgalactosamine 4-sulphate 6-O-sulfotransferase/B-cell recombination-activating genes-associated gene protein)	Sulphate transfer to C-6 hydroxyl group of the GalNAc 4-sulfate residue of chondroitin sulphate A and forms chondroitin sulphate E containing GlcA-GalNAc(4,6-SO_4_) repeating units	(*52*-*54*)
CHST - carbohydrate sulfotransferase. GlcNAc - N-acetylglucosamine. GalNac - N-acetylgalactosamine.		

In summary, literature and other data suggest that CHST are involved in lymphocyte trafficking and homing to inflamed tissue *via* enhanced biosynthesis of selectin L and mucin like glycans (CHST1-4, CHST7). Carbohydrate sulfotransferases 3 and 7 are also expressed in the nervous system where, among others things, they enable the neural cell mobility. Due to its expression in human natural killer-1 cells, CHST10 is also expected to be involved in the immune response. In response to chronic inflammation and other causes of tissue injury, macrophage and fibroblast CHST15 expression is increased and mediates tissue remodelling and fibrosis development. In addition to their roles in inflammation, CHST3, CHST6 and CHST11-14 have a critical role in cartilage and cornea formation. Other CHST are more restricted to specific organs. Thus, CHST5 is expressed almost exclusively in intestines while CHST8 and CHST9 are expressed in the pituitary gland where they catalyse biosynthesis of glycoprotein hormones thyrotropin and lutropin.

Due to their role in cartilage and bone formation and migration of inflammatory and cancer cell, some authors implied that CHST may be used in diagnostics of connective tissue disorders, cancer and inflammations (*10*-*12*). Besides, CHST may also be used for the risk stratification and prognosis (*13*). In addition to the diagnostic and prognostic applications, development of different therapeutic applications in which CHST serve as the molecular targets is underway (*2*, *9*). In summary, the established role of CHST in a wide range of pathological processes makes them a promising candidate for diagnostic applications. Therefore, the aim of this study was to provide an overview of emerging diagnostic and prognostic applications of CHST in different disease conditions, primarily congenital connective tissue diseases and malignant diseases. The following topics on emerging CHST diagnostic applications were covered: application in congenital connective tissue disorders, in cancer, in risk stratification of infectious diseases and pharmacogenomics. The review ends with the laboratory aspects of the reviewed studies and limitations and future prospects.

## Research strategy

**Figure 1:**
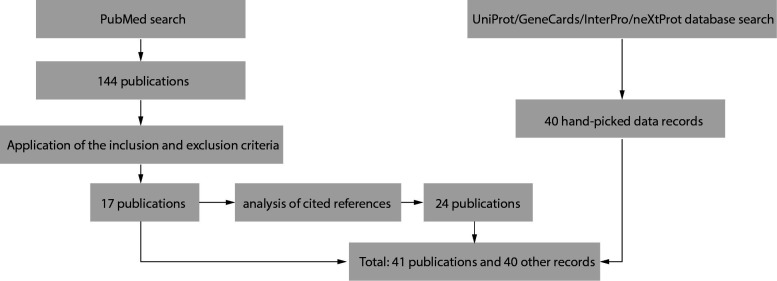
Flow chart of the study selection process. Keywords used for the PubMed search: “carbohydrate sulfotransferase”. Exclusion criteria were: studies using animal models or studies on therapeutic applications. The inclusion criterion was: clinical diagnostic study.

First, a PubMed (National Institutes of Health, USA) search for the publications on CHST diagnostic and prognostic applications was performed. The search was performed using the keywords “carbohydrate sulfotransferase” and it resulted in 144 publications (Figure 1). The following exclusion criteria were used to filter out diagnostically irrelevant records: studies using animal models or studies on therapeutic applications. A detailed analysis of these publications showed that only 17 records met the following inclusion criterion: clinical diagnostic study. Among references cited in these 17 publications, 24 publications meeting the inclusion and exclusion criteria were found, making a total of 41 PubMed records. This unexpected outcome was a result of non-standard CHST nomenclature and diagnostic studies that involved many diagnostic parameters (Table 1). These 41 records were divided into the following categories: congenital connective tissue disorders, cancer, infectious diseases and pharmacogenomics. For proper CHST characterization and classification, additional 40 entries from UniProt, GeneCards, InterPro, and neXtProt databases dealing with CHST genetic and biochemical properties were used in the introductory part of the review what makes a total of 81 records/data entries (Figure 1).

## Diagnostic applications in congenital connective tissue disorders

Mutations and abnormalities in *CHST* genes are directly related to the development of some congenital connective tissue disorders. Pathogenic variants of *CHST* genes cause skeletal dysplasia, chondrodysplasia, and autosomal recessive multiple joint dislocations (ARMJD) (*55*, *56*). Various research studies unveiled the role of CHST3 in congenital skeletal diseases (*55*). Jenniskens *et al.,* Brown and Eames, and Ranza *et al*. confirmed that mutations in *CHST3* caused development of AJMRD and chondrodysplasia with multiple dislocations (*56*-*58*). Sear *et al*., using the *CHST3* gene sequencing, uncovered the new homozygous duplication c.407 426dup (p.Thr143Cysfs*80) in one form of skeletal dysplasia (*59*). Kausar *et al*. showed that *CHST3* mutations cause spondyloepiphyseal dysplasia with joint dislocation, short stature and scoliosis. Clinical evaluations were done on three disorder affected Pakistani families. In-depth mutation analysis was necessary to determine their pathogenicity. The study unveiled biallelic variants c.590 T > C;p.(Leu197Pro), c.603C > A;p.(Tyr201Ter) and c.661C > T;p.(Arg221Cys) of the *CHST3* gene (NM_004273.5) in all families. Carbohydrate sulfotransferase 3 deficiency manifesting as spondyloepiphyseal dysplasia was also described by Thiele *et al.,* (*60*). Moreover, Waryah *et al*., by using the whole exome sequencing (WES), identified a novel point mutation (c.802G > T;p.Glu268*) in *CHST3* gene associated with spondyloepiphyseal dysplasia and hearing loss in Pakistani relatives (*61*).

Hennet *et al*. suggested that two more congenital connective tissue diseases are associated with defective carbohydrates sulfation caused by mutations in *CHST6*, and *CHST14* genes: macular corneal dystrophy is caused by the *CHST6* deficiency while some mutations of the *CHST14* gene cause a new form of the Ehlers-Danlos syndrome (N = 19 and N = 11, respectively) (*62*-*64*). Finally, the prognostic significance of *CHST* genes mutations in the connective tissue disorders was shown in study by Blanco *et al.* (*65*). The aim of the study was to find a predictive model based on genetic polymorphisms and clinical variables that would be used as a genetic prognostic model of the primary knee osteoarthritis (OA). Interestingly, unlike other studies of congenital connective tissue diseases described here, in which whole blood samples were used to collect genetic material, saliva or even serum samples were used in this study. Authors included the polymorphisms located in the candidate genes previously known to be implicated in the OA development, generating the list of approximately 774 single nucleotide polymorphisms (SNPs). Results of their multivariate logistic regression analysis indicated that the polymorphism located in the *CHST3* gene (rs874692) was significantly associated with knee OA progression (N = 220, odds ratio (OR) = 2.36, P = 0.017). Addition of 23 gene polymorphisms, including the mentioned *CHST3* polymorphism, to the predictive model containing only clinical variables improved its performance measured by receiver-operating-curve (ROC) area-under-curve (AUC) from 0.62 to 0.82 (sensitivity = 93%, specificity = 55%, positive predictive value = 57%, negative predictive value = 92%). It was also found that *CHST11* gene polymorphism (rs835487) is associated with prevalence of hip OA (*65*).

## Cancer diagnostics, risk stratification and prognosis

Various members of CHST family have been implicated as contributors to carcinogenesis and tumour progression and as such have prognostic potential in various cancer types (*66*-*69*). Aberrant activation of *CHST* genes is linked to pathological processes like tumour initiation and progression. Carbohydrate sulfotransferase and chondroitin sulphate are also involved in metastasis at various sites. It has been shown that CHST3, CHST7, CHST11-13 and CHST15 have functional relevance and prognostic potential in various cancer types (*66*). For example, study of normal and malignant human stromal cells and prostate epithelial suggested that CHST15 overexpression leads to non-canonical wingless/int-1 (WNT) signalling activation, a characteristic hallmark of cancer (*70*). Similar result has been found in the glioblastoma (GBM) case where the CHST12 tissue expression was also associated to the WNT signalling (*71*). A recent study has revealed that CS and CHST also play a significant role in the metastatic spread of tumour cells through adhesive properties modulation (*72*). The study has shown that CHST11, CHST12 and CHST15 messenger ribonucleic acid (mRNA) tissue expressions in the malignant ovarian tumours were increased compared to the expressions in the non-malignant ovarian tumours (N = 95, P < 0.05 for each gene expression).

In the context of malignant diseases, the most thoroughly described are diagnostic applications of CHST7 and CHST15. Carbohydrate sulfotransferase 7 controls the CSPG concentrations, which contribute to metastatic processes and carcinogenesis (*73*, *74*). Deoxyribonucleic acid methylation of the *CHST7* gene, determined by the methylation-sensitive high-resolution melting (MS HRM), was linked to the pituitary tumour and colon cancer development (*75*, *76*). Hypermethylation of CHST7 found in pituitary adenomas was associated with tumour proliferation (N = 106, P = 0.026) (*75*). A similar result was obtained in the evaluation of CHST7 role in colorectal cancer. The results indicate that *CHST7* gene hypermethylation in the white blood cells is associated with the increased risk of colorectal cancer (N = 432, OR = 4.45, P < 0.001) (*76*). Significant differences in *CHST7* gene expressions were detected in malignant lung tissue samples as compared to the adjacent non-malignant tissues (N = 46, P < 0.001) (*77*). In this regard, possible application of the serum CHST7 concentration in differentiation of non-malignant pulmonary inflammations and non-small cell lung carcinoma (NSCLC) has been shown (N = 125, AUC = 0.85, sensitivity = 78%, specificity = 75%) (*73*). These results were preceded by the univariate statistical analyses revealed significant differences in the peripheral blood mononuclear cells CHST7 expressions between NSCLC and non-malignant pulmonary diseases (*73*, *74*).

The CHST15 overexpression, determined by immunohistochemically staining, has been reported in pancreatic ductal adenocarcinoma (*78*). Presented data show a significant negative association between the disease-free survival and CHST15 expression (N = 36, hazard ratio = 9.456, P < 0.001). Although the study was unable to show which cell types were CHST15 positive, it provided insight into the possible SULT application as target for pancreatic cancer treatment. A similar study showed that CHST15 expression could be detected in the pancreatic cancer cells cytoplasm and fibroblasts in the cancer stroma. High CHST15 expression in the cancer stroma led to worse overall survival as compared to low CHST15 expression associated with the higher incidence of immature fibrosis (N = 64, P = 0.02) (*67*).

In addition to NSCLC and pancreatic cancer, the diagnostic properties of CHST were evaluated in other cancers. Oliveira-Ferrer *et al*. compared the mRNA expression of the different CHST in malignant and non-malignant ovarian tumours (*72*). The study results revealed that there was significantly higher mRNA expression of CHST11, CHST12, CHST15 and CHST13 compared to non-malignant tumours (*72*). Western blot analysis of tissue homogenates showed high CHST11 expression, which was individually associated with shorter progression-free survival in ovarian cancer (N = 216, P = 0.027) (*72*). Wang *et al*. have shown that CHST12 is highly expressed in GBM (*71*). They analysed GBM tissue by immunochemical methods and quantitative real-time polymerase chain reaction (qRT-PCR). Univariate and multivariate regression analyses showed CHST12 overexpression in comparison to the adjacent healthy tissue (N = 60, P < 0.003 and P < 0.021, respectively). Results presented in this study also indicate potential prognostic role of CHST12 in GBM development and progression.

## Risk stratification in infectious disease

It is well known that certain types of cancers are associated with viral infection. Chronic hepatitis B virus (HBV) infection is a major risk factor for hepatocellular carcinoma (HCC) and about 50% of HCC are HBV positive. Carbohydrate sulfotransferase 4 is expressed abnormally in different cancers and plays an integral role in lymphocyte homing. It has been shown that its downregulation promotes HBV expression and appearance of hepatocellular carcinoma associated with hepatitis B virus (HBV-HCC). On the other hand, increased CHST4 expression is needed for macrophage, CD4+ cell, neutrophil, and dendritic cell recruitment that inhibits HBV-HCC progression. Zhang *et al*. revealed that in HBV-HCC CHST4 tissue expression was downregulated as compared to the normal tissues (N = 242, P < 0.001) which may promote HBV expression and malignant behaviours in HBV positive HCC. Their results also indicate a possible role of CHST4 in CD4+ T cells, macrophages, dendritic cells and neutrophils recruitment into tumour microenvironment which may lead to inhibition of tumour progression. To conclude, results of their analysis suggest that CHST4 expression could be recognised as a tumour suppressor in HBV positive HCC and potential diagnostic and therapeutic target. It has been found that high CHST4 expression is favourable and independent prognostic factor of overall survival in patients with HBV-HCC (N = 242, P = 0.002) (*12*).

During the past few years, the COVID-19 pandemic represented a unique challenge. Developing more accurate diagnostic and prognostic tools was an important task. With that goal in mind, Tzankov *et al*. evaluated the roles of different CHST in human vascular smooth muscle cells by qRT PCR technology. They found that increased CHST11 and CHST15 activity may lead to severe lung pathology in coronavirus disease 2019 (COVID-19) patients. Carbohydrate sulfotransferase 11 mRNA expression was increased by 3.1 times (N = 9, P < 0.001) and CHST15 expression was 2.1 times (N = 15, P < 0.001) higher in the vascular smooth muscle cells of COVID-19 patients that had severe lung manifestations (*79*). These changes in CHST expression may increase the generation of chondroitin 4-sulfate and chondroitin sulphate E what, with N-acetylgalactosamine-4-sulfatase activity being suppressed, may cause a vicious cycle that ends in refractory respiratory failure.

Severe inflammations may also induce life-threatening thrombotic storms (TS). By using WES and whole blood samples, Nuytemans *et al*. found pathogenic gene variants of *CHST3*, *CHST12*, and *CHST15* genes, in more than 30% of TS affected patients (N = 26, P < 0.05 for all gene variants) (*80*). Taken together, CHST expression and polymorphisms represent new risk factors for infectious disease complications.

## Pharmacogenomics

Genomic information is used to predict individual drug response. Yorifuji *et al*. conducted a comprehensive genomic association study using whole blood samples and DNA microarray and found that two SNP in *CHST3* and *CHST13* genes were significantly associated with bosentan-induced toxicity (*81*). The study extracted 16 SNPs (N = 69, P < 0.05 for each SNP) using the Jonckheere-Terpstra trend test and multiplex logistic analysis. From all identified SNPs, two genes *CHST3* and *CHST13*, which are responsible for PG sulfation were significantly associated with the bosentan-induced liver injury. Researchers constructed a predictive model for bosentan-induced liver toxicity using these two SNP and two non-genetic factors: AUC was 0.89 (sensitivity = 83%, specificity = 86%). The findings demonstrate that the *CHST3* and *CHST13* alleles are much more common in individuals with increased aminotransferases and liver impairment after bosentan therapy than in other patients (*81*).

## Laboratory aspects of the reviewed studies

A wide variety of molecular diagnostics and immunochemical methods including WES, MS HRM, qRT PCR, gene expression and DNA microarrays, Western blotting, immunoassays and immunostaining were described in the reviewed studies. Western blotting, immunoassays and immunostaining are well known methods already available in clinical laboratories while, thanks to COVID-19 pandemic, qRT PCR entered many clinical laboratories relatively recently. Whole exome sequencing and MS HRM are the “research use only” but promising molecular diagnostics methods used for detection of mutations and estimation of gene methylation. Status of gene expression and DNA microarrays in clinical laboratory diagnostics is still unclear. Although not frequently used in the routine clinical laboratory, this technology underlies many commercial tests used for cancer risk stratification and prognosis.

It is interesting to notice that, besides the initial method development described by Paul *et al.*, no clinical evaluations of serum CHST diagnostic performance have been performed. Instead, gene or protein expressions has been measured (*3*). Since photometric enzyme activity assays are compatible with widely available automatic chemistry analysers, the adaptation of CHST activity assays for automatic analysers would enable wider and more reliable diagnostic use. Detection of enzyme activities is also compatible with microscopic tissue analyses. Development of CHST activity based microscopic analysis is expected to improve availability of these analyses.

## Limitations and future prospects

According to the inclusion and exclusion criteria, studies covered by this review include clinical CHST test developments and evaluations or at least, suggestions on possible CHST clinical applications. Some of the selected studies rely more on preclinical data what may only imply the CHST clinical utility (*10*-*12*, *57*, *58*). These implications should be taken with caution since they still lack a proper clinical evaluation. Clinical utility of presented qualitative molecular diagnostic tests relies mostly on anecdotal evidence what is inevitable since these tests are aimed at the rare congenital diseases for which large patient’s cohorts cannot be gathered. However, in most cases, quantitative tests described in the selected studies were evaluated using the ROC or survival analysis which provide more reliable diagnostic and prognostic data, respectively. Unfortunately, the number of participants enrolled in these studies varies from a few dozens to several hundreds. Larger cohorts are needed for the more reliable diagnostic evaluation. Based on the ROC analysis performed in a single laboratory, a significant diagnostic accuracy has been assigned to the serum CHST7 concentration in differentiation of all stages of NSCLC from the non-malignant pulmonary inflammations (*73*). However, an attempt to reproduce these results on the stage 1 and 2 of NSCLC failed, presumably due to variable reagent quality (results not shown). This indicates the need for multicentre and independent evaluation and also the need for robust analytical methods.

The presented evidence coming from the early clinical studies shows CHST diagnostic potential. However, there is a gap between routine clinical usage of CHST tests and presented results. There is an evident lack of large multicentre diagnostic evaluations and robust analytical procedures. Without these reviewed CHST tests will not gain wider clinical use in foreseeable future.

## Conclusion

A wide variety of molecular diagnostics and immunochemical tools including WES, MS HRM, qRT PCR, gene expression microarrays, Western blotting, immunoassays and immunostaining were used in CHST diagnostic applications research. Selected studies have shown the lack of CHST activity in some congenital connective tissue disorders and CHST overexpression in different malignant tissues. Gene mutation analysis of *CHST3, CHST6* and *CHST14* should be performed whenever there is suspicion of spondyloepiphyseal dysplasia, Ehlers-Danlos syndrome, macular corneal dystrophy and ARMJD, respectively. On the other hand, CHST7 expression is NSCLC candidate biomarker, while *CHST7* gene hypermethylation in white blood cells is associated with increased risk of colorectal cancer. Increased tissue expressions of CHST11, CHST12 and CHST15 were shown to be unfavourable prognostic factors of ovarian cancer, GBM and pancreatic cancer, respectively. Recently, some interesting diagnostic applications of CHST also emerged in infectious diseases and in pharmacogenomics. These facts grant dynamic future development of CHST diagnostic and prognostic applications. However, a review of literature calls for caution. More reliable assessment of CHST diagnostic applications is needed. That assessment should be based on the clinical studies involving large and well defined cohorts of patients and use of robust analytical procedures.

## Data Availability

No data was generated during this study.
